# Implementation of the drive-through strategy for COVID-19 vaccination: an experience report

**DOI:** 10.1590/1980-220X-REEUSP-2021-0397en

**Published:** 2022-05-16

**Authors:** Letícia Yamawaka de Almeida, Jessica Domingues, Talita Rewa, Daniela Baptista Novaes, Adriana Aparecida Alves do Nascimento, Daiana Bonfim

**Affiliations:** 1 Hospital Israelita Albert Einstein, Centro de Estudo, Pesquisa e Prática em APS e Redes, São Paulo, Brazil.

**Keywords:** COVID-19, Immunization Programs, Health Planning, Nursing, Public Health Surveillance, Primary Health Care, COVID-19, Programas de Inmunización, Planificación en Salud, Enfermería, Vigilancia en Salud Pública, Atención Primaria de Salud, COVID-19, Programas de Imunização, Planejamento em Saúde, Enfermagem, Vigilância em Saúde Pública, Atenção Primária à Saúde

## Abstract

**Objective:**

To describe the experience of implementing a satellite vaccination unit in a drive-through system during a campaign against COVID-19.

**Method:**

This is an experience report carried out in a drive-through vaccination satellite unit. The study development was guided by the triad structure-process-results, proposed by Donabedian.

**Results:**

The unit was structured in a soccer stadium, allowing it to serve large audiences safely. Care flow occurred in stages and professionals were organized by sectors, with emphasis on the nursing team’ work. Initially, screening was performed; later, users went to the registration sector, and, finally, they were forwarded to the application station. The unit also had emergency sectors, a cold chain, space for professionals and a Basic Health Unit as a point of support. In 25 days of operation, 9698 doses were administered, with 1.8% of doses lost.

**Conclusion:**

The implementation of this system required planning, structure, process development and intense team articulation, with emphasis on the fundamental and strategic role of nurses in different points of action and leadership.

## RESUMO

**Objetivo:**

Descrever a experiência de implementação de uma unidade satélite de vacinação em sistema *drive-through,* durante a campanha contra COVID-19.

**Método:**

Trata-se de um relato de experiência, realizado em uma unidade satélite de vacinação em sistema *drive-through*. O desenvolvimento do estudo foi norteado pela tríade estrutura-processo-resultados, proposta por Donabedian.

**Resultados:**

A unidade foi estruturada em um estádio de futebol, permitindo o atendimento de grandes públicos de forma segura. O fluxo de atendimento ocorreu por etapas, e os profissionais foram organizados por setores, com destaque para atuação da equipe de enfermagem. Inicialmente, realizou-se a triagem, posteriormente, o usuário dirigia-se ao setor de cadastramento, e, por fim, era encaminhado à estação de aplicação. A unidade contava também com os setores de urgência e emergência, rede de frio, espaço para os profissionais e uma Unidade Básica de Saúde como ponto de apoio. Em 25 dias de atuação, foram administradas 9698 doses, com 1,8% de doses perdidas.

**Conclusão:**

A implementação deste sistema exigiu planejamento, estrutura, desenvolvimento de processos e intensa articulação em equipe, com destaque para o papel fundamental e estratégico do enfermeiro em diferentes pontos de atuação e liderança.

## RESUMEN

**Objetivo:**

Describir la experiencia de implementación de una unidad de vacunación satélite en un sistema drive-through, durante la campaña contra el COVID-19.

**Método:**

Se trata de un relato de experiencia, realizado en una unidad satélite de vacunación drive-through. El desarrollo del estudio estuvo guiado por la tríada estructura-proceso-resultados, propuesta por Donabedian.

**Resultados:**

La unidad se estructuró en un estadio de fútbol, lo que le permitió atender grandes audiencias de manera segura. El flujo de la atención ocurrió por etapas, y los profesionales fueron organizados por sectores, con énfasis en el trabajo del equipo de enfermería. Inicialmente se realizaba el cribado, posteriormente el usuario se dirigía al sector de registro y finalmente se enviaba a la estación de aplicación. La unidad también contó con sectores de urgencia y emergencia, cadena de frío, espacio para profesionales y una Unidad Básica de Salud como punto de apoyo. En 25 días de operación, se administraron 9698 dosis, con un 1,8% de dosis olvidadas.

**Conclusión:**

La implementación de este sistema requirió planificación, estructuración, desarrollo de procesos y una intensa articulación de equipos, con énfasis en el papel fundamental y estratégico de los enfermeros en diferentes puntos de acción y liderazgo.

## INTRODUCTION

The race to produce vaccines against COVID-19 is a historic milestone, with human clinical trials (phase I) starting in March 2020^(1)^ and approval of candidate vaccines for emergency use in December 2020^(2)^. However, this advance required global responses and efforts that matched the pace of vaccine development and, at the same time, ensured the vaccine acquisition, supply and distribution on a large scale, aiming at collective immunity^(2,3)^.

Thus, a new challenge^(3)^ established to face the pandemic was characterized by the vaccine operationalization, distribution and administration in a strategic, fast, safe way and with population impact. In this sense, some successful experiences of vaccination campaigns were recorded, especially among high-income countries^(4,5)^.

The results obtained in the successful experience of Israel, for example, demonstrated the importance of combining “facilitating” factors at different levels. Such factors involve characteristics (political, geographic and demographic) of the country’s health system, in addition to the efforts made directly to face the pandemic^(4)^.

In general, strategies used in the first mass vaccination programs against COVID-19 include prioritizing vulnerable groups, use of large public spaces quickly reorganized to accommodate vaccination centers, implementation of drive-through system and vaccination points in nursing homes, hospitals, clinics and churches. In addition, the involvement of volunteers (military and civilian) and communication measures aimed at raising awareness and acceptance of vaccination were strategies considered in the implementation of promising initiatives in high-income countries^(5)^.

It is considered, however, that the progress achieved in these countries does not reflect the global reality, nor is it characterized as a trend in low- and middle-income scenarios. In Brazil, despite the existence of the Brazilian National Immunization Program (PNI – *Programa Nacional de Imunizações*), internationally recognized for its successful trajectory in the eradication and control of vaccine-preventable diseases^(6)^, the country has faced obstacles in the implementation of control measures in the current health situation, especially with regard to the coordination of immunization actions.

Strictly speaking, Brazilian municipalities have organized themselves into different strategies for the implementation of vaccination, such as drive-through, which, among other methods, has been suggested as one of the possible effective strategies for mass vaccination^(7)^.

Considering this scenario, the present study aimed to describe the experience of implementing a satellite vaccination unit in a drive-through system, during the campaign against COVID-19.

## METHOD

### Design of Study

This is an experience report, carried out in a drive-through vaccination satellite unit. Experience reports are characterized as texts that, by sharing details of situations, procedures and strategies experienced/used in care, pedagogical or scientific daily life, they provide elements for reflection among peers and enable their application in other scenarios. To do so, they must essentially answer the following questions: why? When? Where? How? Who?^(8)^.

### Scenario

The experience described was developed in southern São Paulo, SP, through an agreement that has existed for 20 years between the Municipal Health Department and the *Sociedade Beneficente Israelita Brasileira Hospital Albert Einstein*, an institution in which the researchers work.

The municipal immunization campaign’s operational plan, aimed at wide vaccination coverage, had fixed, mobile and drive-through vaccination health centers. Such health centers, distributed in all regions of the city, were organized in health units, clubs, churches, shopping centers, universities, race tracks, parks and soccer stadiums^(9)^.

A drive-through system, which makes up the campaign structure, is characterized as a large vaccination center that makes it possible to serve the public without the need for individuals to leave their cars. During the period of development of this study, the municipality had approximately 25 vaccination stations in this system^(9)^.

The services were carried out to the public, according to the instructions provided by the city hall^(9)^. The activities were carried out from March 2 to May 8, 2021, intermittently (totaling 25 days of service), considering municipal guidelines on priority groups and changes in target audience. The service was open from Monday to Saturday (including some holidays) from 8 a.m. to 5 p.m. To close the activities and close the unit, the team had support from Traffic Engineering Company.

### Process Description

The results described in this study are a result of the participatory, structured observation of researchers who worked in the planning and leadership of the satellite unit work process implementation. Moreover, an analysis of institutional documents was carried out. The observation process took place from March to May 2021.

### Data Analysis

To describe the implementation process of this unit, the structure-process-results triad proposed by Donabedian was used^(10)^. According to the author, the structure category refers to the environmental attributes in which care takes place. It is, therefore, material resources (facilities and equipment), human resources (professional team quantity and qualification) and organizational structure. The process category concerns the actions performed by professionals and users when offering and receiving care, respectively. Finally, the result category is characterized as the effects of assistance provided to users’ health^(10)^.

It is noteworthy that the use of this framework was considered for the present proposal as it allows a systemic and structured view of the material resources, processes/flows implemented and the outcomes achieved with the experience described.

### Ethical Aspects

This experience report originated from the activities developed in research that seeks, among its specific objectives, to describe professionals’ activities and the work process organization of family health teams. The research was approved by the Research Ethics Committee of *Hospital Israelita Albert Einstein* (Opinion 4,746,712/2021). It is noteworthy that the study was conducted in accordance with Resolution 466/2012, which regulates research with human beings.

## RESULTS

### Structure Category

The structure planning was carried out as a matter of emergency, since the interval between the request to open the site and the start of activities was a period of four days. Thus, at first, the partner institution established a team responsible for planning and organizing activities at the satellite unit.

This team, composed predominantly of leaders from the institutional technical support and quality area of the aforementioned institution, carried out on-site visits and meetings for structural planning. This fact involved significant internal communication with the Municipal Health Department health surveillance unit, to align the prescription of doses, supplies, accountability and reports. Aspects related to the satellite unit physical and material structure (facilities and equipment), human resources and organizational structure were described below.

### Physical and Material Structure

This satellite unit was implemented in a soccer stadium, requiring adaptations in the structures provided by the club to organize and operationalize service flow to users, storage of inputs/materials and points of attention aimed at professionals’ well-being. The use of this space made it possible to serve large audiences, with reduced exposure among users, companions and the health team.

One of the fundamental aspects included in the planning stage concerns the acquisition of equipment such as tables, chairs, medication carts, emergency materials, freezers, minibar, notebook, tablets (contracted mobile internet plan) for use in registering the doses applied, office supplies, trash cans and informational banners/banners. It is noteworthy that all these resources were provided within 24 hours.

**Figure 1.**
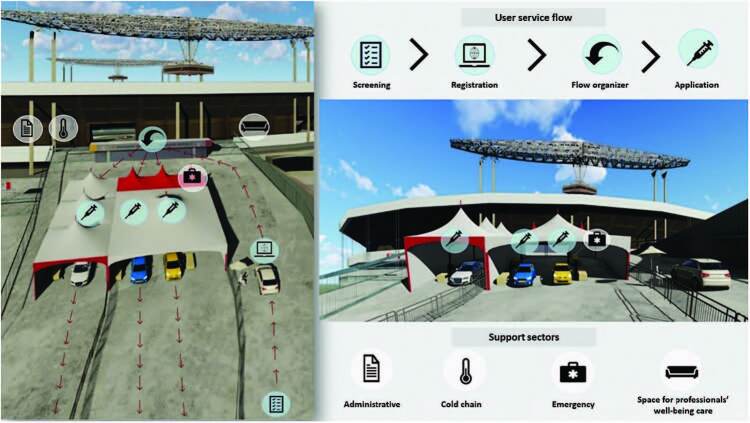
Physical structure, sectors and operationalization of user service. São Paulo, SP, 2021.

The project was designed for the simultaneous service of up to nine cars, distributed in three zones and different application stations (three tables in each zone). Such stations were opened or closed according to the volume of demand. To house the application sector, 10m^2^ tents were installed, with thermal comfort, side closure and permanent fixation on the ground as a safety measure. Figure 1 shows the physical structure, sectors and operationalization of service to users.

As can be seen, user service was organized in one of the stadium’s access ramps. This is a large space with direct access to the public road. These conditions allowed the operationalization of the one-way service flow organized into sectors, according to the different stages that comprise the vaccination process in a drive-through system.

The screening, registration and flow organization sectors had chairs and umbrellas for the comfort of professionals. In the application sector, tables were planned with distance and distribution of materials and supplies, aiming at user and professional safety. It should be noted that this area also had the emergency sector, provided with resources to assist all intercurrences. Information on the materials and inputs used in these sectors was made available as supplementary material.

A strategic sector for the vaccination process refers to the cold chain. This sector was structured in a corridor located at the end of the access ramp in a covered area (masonry) and flat surface. For the proper cold chain maintenance, refrigerators were set up for storage and conservation, support furniture (tables), thermal boxes for packaging and transporting vaccines to the application stations. It is noteworthy that a Basic Health Unit (BHU) located close to the stadium was established to support vaccine storage agents during the drive through’s non-operational hours.

In an area close to the cold chain, the administrative sector was located, which had tables, chairs, cabinets, computers connected to the internet and other office supplies, in addition to a white board, hanging on the wall, which indicated the leaders’ names in the period, useful telephone numbers and described the standard operating procedures (SOP), which were available for consultation by professionals.

Finally, spaces for attention to professionals’ well-being were planned and contemplated the food area and decompression space with chairs and cabinets. In the other sectors, coolers and refrigerators with glasses of mineral water were distributed in different parts of the environment. It is noteworthy that, in order to reduce sun exposure during work, umbrellas were installed in the sectors discovered in the area of user service. Furthermore, sunscreen, cap (or similar) and raincoat were offered to professionals.

### Human Resources

In addition to the team responsible for planning and organizing the satellite unit activities, relations with the press, local leaders and work team scheduling, which was configured on an itinerant basis and had professionals (middle and higher education) from BHUs, Psychosocial Care Centers (CAPS – *Centros de Atenção Psocossocial*) and institutional technical support area, were determined to be responsible for conducting the project.

It is noteworthy that the leaders established for the satellite unit were mostly nurses who worked in different institutional positions. To facilitate the recognition of sector leaders among team members, the strategy of identification with colored bands on the leaders’ uniform was used.

**Figure 2.**
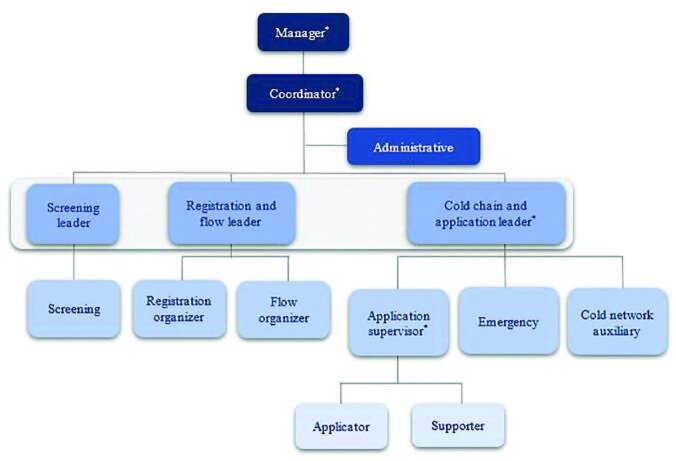
Satellite vaccination unit organization chart. São Paulo, SP, 2021.

Figure 2 shows the vaccination satellite unit organization chart, highlighting the roles performed by nurses.

*Roles performed by nurses.

The description of activities carried out by satellite unit professionals, according to sector and role, was organized in a document and made available as supplementary material.

### Process Category

The processes carried out at the unit were designed considering the steps and procedures involved in vaccination, an action plan to reduce lost doses, definition of roles performed by professionals, preparation of contingency plan to maintain activities in exceptional situations. These aspects were compiled and detailed below. It is worth mentioning that, to outline the flows and strategies of operationalization, the planning team carried out benchmarking in a satellite unit that operated in a drive-through system in another region of the municipality.

### Activities and Flows

Considering that the work team was organized itinerantly and had professionals from different services and professional categories, arrangement of roles was defined according to the professional profile and immunization experience. In order to standardize the actions and ensure best practices in the unit, a training stage was offered on-site to professionals.

In general, the content covered was based on the presentation of the unit organization, flows and organizational chart, guidelines directed to roles developed in each sector and instructions on user and professional safety measures. It is noteworthy that training was replicated whenever new professionals started their activities in the unit.

In the screening sector, professionals approached users to validate the eligibility criteria for vaccination. To assist the team members in this process, a printed form was prepared, in checklist format and made available in the sector. In cases where the criteria were not met, users were guided according to the instructions of the Municipal Health Department in force.

Then, cars were directed to the entrance gate and started the stage called registration, in which a professional carried out the application of a health questionnaire on chronic diseases, allergies, medication use and filling in the information on a tablet in the state information system. At the end, the vaccination card was delivered to users.

Subsequently, a professional in charge of organizing the service flow directed users to one of the stations available in the application sector to perform the procedure. Each station had two professionals responsible for guiding, documenting and administering the vaccine. At the end of this process, users were sent to the exit gate with access to the public road (Figure 2).

In the cold chain area, temperature monitoring, control and recording was carried out, as well as the packaging of all vaccines available in the satellite unit. The operation of activities in this sector was fundamental for carrying out actions in the unit and began before the drive-through opened to the public.

At first, thermal boxes were stabilized to receive the vaccine and, later, they were distributed to the application stations. Moreover, the boxes that were on stand-by were prepared for cases that required immediate replacement, for example, in situations of variation in the temperature of thermal boxes allocated to the stations.

It is noteworthy that the entire cold chain of vaccines was respected, being organized a daily schedule of nursing professionals (nurses assistants and nurses) was organized, who worked specifically in local maintenance and control, checking the temperature in all application stations and in the vaccine chamber.

Considering the global scenario of shortage of vaccines against COVID-19, strict control measures were implemented for the best use of available doses. Such measures contemplated the availability of one or two vials at each station and input replacement was performed after a request from professionals in the application area who, upon receiving new vials of vaccine, delivered the empty vials. At this moment, validation was carried out on the number of doses made with that bottle, as well as the identification of a new one’s opening date and time. In addition to this, the empty bottle was registered and stored for conference at the end of the day.

Vaccine bottle flow between the satellite unit and the support unit was performed daily. At the beginning of each working day, institutional transport sent vaccines to the stadium, under the supervision of a nurse or nursing assistant, responsible for controlling the temperature during the journey. At the end of the day, the same procedure was performed for the storage of vaccines in a BHU. If there were plenty of doses, a support BHU was notified in advance to summon the users enrolled on the waiting list.

### Risk Management

Regarding the safety measures, different actions were organized, namely: notification flow for adverse events; technical complaints about the quality of all materials used; occupational safety visits to assess possible risks to professionals; and carrying out safety rounds called safety huddle.

The safety huddle was led by the nurse responsible for the application sector and was configured as the first activity performed by the team in the period. Professionals from different sectors met in a circle format and briefly recalled the important points foreseen for the development of activities that day.

Among the topics routinely addressed, the following stand out: age group and eligible group; professional and user safety aspects; process of conference and confirmation of data with users; correct procedure (preparation, route, volume, site) of vaccine administration; temperature control of thermal boxes; map release, scheduling and lot; adequate approach to users and family members; role of reference and supervision of nurses and other leaders.

Also, it was emphasized to the nursing assistants that, if they felt uncomfortable or insecure to perform any activity, they should signal the supervisor nurse in the area. Despite being infrequent, in these situations, nurses took over the case management.

It is worth mentioning that, due to the feeling of insecurity present in users regarding the procedure of administering the immunizing agent, the applicators were constantly oriented on the importance of maintaining transparency throughout the vaccine preparation and administration.

In this sense, some essential steps were standardized for the moment of application, such as presentation of bottle, indication of producer laboratory, expiration date, dose aspiration and, after administration, presentation of empty syringe. Given this scenario, it was necessary to add a support professional (identified as a supporter) to the application team to guarantee a transparent and safe process for all involved.

Another protection measure instituted refers to user/vehicle positioning during the procedure. Professionals were instructed to ask users to turn off the car and remain seated in the vehicle’s seats, signaling that the circulation of users in the application area was not allowed. Additionally, the vehicle driver was asked to verify that the handbrake was activated.

As for the aspects related to the management plan for the waste generated at the unit, the process established aimed to ensure proper management and safety at all stages, from packaging to final destination.

### Result Category

**Figure 3.**
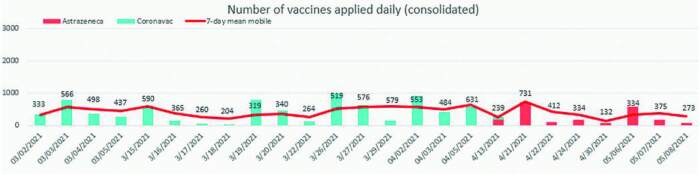
Number of doses applied according to vaccine and period. São Paulo, SP, 2021.

In 25 days of operation, 9,698 doses of immunizing agents were administered, as can be seen in Figure 3. It is noteworthy that the significant increase in the frequency of visits in certain periods corresponds to the dates of inclusion of new age groups in the municipality’s vaccination schedule.

The percentage of missed doses was 1.8%. However, it is noteworthy that the registration of missed doses was performed when the bottle resulted in a smaller volume of doses than indicated on packaging. This condition could be related both to failures of the producing laboratory and to the use of common syringes and needles that are not of low dead volume, with discreet retention of the dose volume in the syringe nozzle and needle barrel. It is indicated that, during the entire period of activities, there were no losses of doses due to any other condition.

Among the challenges present in the drive-through implementation is the active team composition, made up of professionals from different units who, for the most part, did not know each other. In addition, the teams worked on an itinerant basis, i.e., professionals performed a rotation system, according to availability (considering the need for its original unit), culminating in new (daily) arrangements in the team and the need for constant training.

Workload both for the leadership work and for those who were in direct contact with the public, especially on days when new age groups were included in the calendar, it was perceived as another challenging factor in the daily work of professionals who worked at the unit. The need to stand for long periods, exposure to the weather, walking for long distances, regardless of the sector of activity, were also difficulties faced by the team.

Nursing professional sizing was another challenge experienced due to the scarcity of studies in the national literature available for the calculation and planning of professionals in situations of large-scale vaccination campaigns. Thus, due to the absence of parameters referring to the drive-through modality, the schedule of professionals was organized considering the installed capacity of 1,000 doses in a period of eight hours, to be carried out in up to nine application stations. In other words, the calculation was performed by means of proportion. However, considering that sizing must be dynamic, with the work process, it was constantly updated.

A very peculiar characteristic related to the current health context concerns the constant contact and exposure of professionals to communication vehicles and approaches from family members. This new condition, in certain circumstances, compromised the natural conduct of activities, in addition to embarrassing some team members.

Thus, some strategies were adopted in order to mitigate the effects of this stressor, such as warning team members in advance about the media’s participation in that period and identifying professionals who felt comfortable to be monitored. Furthermore, constant family approaches (requesting photos and recordings) impacted time and work dynamics.

Some common operational issues in vaccination campaigns have become challenging due to the volume of demand, namely systematic recording and reporting of doses applied, stock control, vials consumed and strict criteria to ensure synchrony between the time of completion of activities at the drive-through and the number of doses/vials opened at the stations, in order to avoid loss of immunobiological doses agents. Furthermore, in periods of instability in the registration platform, information registration was performed manually and, later, transcribed and documented in the system.

A highlight is related to team willingness and positive manifestations in the face of gratitude expressed by users and family members when receiving the vaccine doses, promoting a climate of hope and victory in the face of obstacles and difficulties experienced since the beginning of the pandemic, especially among health professionals, thus reflecting on the strengthening and cohesion of teamwork.

## DISCUSSION

Large-scale vaccination efforts are being carried out all over the world and some mathematical models^(7)^, described in the literature, have sought to support this implementation. However, information on the first mass vaccination strategies against COVID-19 identified globally comes from policies and guidelines focused on high-income countries^(5)^.

This is, therefore, the first known study that describes the implementation of a drive-through vaccination satellite unit, in an emerging country with universal health coverage, during the campaign against COVID-19. Under the light of the structure-process-result triad^(10)^, aspects of planning, infrastructure conditions and human resources, activities offered as well as challenges and good practices were presented, providing subsidies for managers in addition to expanding the debate in scientific literature.

Regarding the structural aspects, it was observed that the pandemic scenario required major adaptations for a safe conduct of the immunization campaign. As indicated in the results of this study, the satellite unit implementation took place in a soccer stadium, supporting the World Health Organization (WHO) recommendations^(11)^ and following the trends of campaigns in developed countries with a drive-through system^(5)^.

It is noteworthy that vaccination strategies in environments outside the vaccine rooms require rigorous planning so that their operation is carried out efficiently and safely. The success of the Israel campaign’s initial implementation phase, for instance, was linked to the organizational, logistical and information technology capabilities of health providers as well as the country’s experience in planning and implementing rapid responses to large-scale emergencies^(4)^.

It is worth noting that the historical trajectory of PNI^(6)^ in Brazil enabled professionals, especially nurses, to accumulate knowledge and develop skills for the management of large vaccination campaigns, acting in this scenario (unprecedented and with a lack of references), in an expressive way, in positions of leadership and operation, in an assertive way.

With regard to the immunization process, consolidated in the scope of nursing practices, the experience of countries such as Israel also indicates that the team of community nurses qualified to conduct the campaign was a factor that facilitated achieving positive results^(4)^. Thus, the findings of this study, by emphasizing the nursing team’s work and strategic roles (management and care) exercised in the vaccination service, endorse the idea and give visibility to the potential of nursing contributions in the management of a serious public health problem.

Added to this, the expansion of Primary Health Care (PHC)^(12)^, through Family Health Strategy, enabled the incorporation of a multidisciplinary team that, faced with the challenge of operationalizing the vaccine drive-through, acted in an integrated way, including different health professionals, of medium and higher level, who continuously participated in the activities in the different satellite unit sectors. In this regard, the proposed model became a space for strengthening integrated teamwork, in addition to highlighting the potential of PHC professionals in dealing with health emergencies.

It was observed that the pandemic context also implied the need to reorganize operational issues and activities already well defined in vaccine rooms and previous campaigns. Apparently, the sum of some characteristics of the current scenario, such as the short-term development of the vaccine, the dissemination of fake news and the wave of vaccine hesitancy weakened, to some extent, the population’s confidence in the vaccination process^(13, 14, 15, 16)^.

In this way, aspects aimed at increasing safety in the process were more densely incorporated into the campaign environment, which are discussed more frequently in a hospital environment, such as encouraging users and family participation in the process, through team inquiry about information regarding the administered vaccine, acting as barriers to minimize risks of error in the vaccine administration^(17)^.

Another very particular experience of the current scenario, which somehow impacted the organization of activities offered in the satellite unit, refers to the photographic records of the procedure performed by users, family members and media vehicles. If, on the one hand, this condition demonstrated the social relevance of vaccination as a symbol of overcoming, citizenship and self-care, on the other hand, it was presented as a form of control and inspection in the face of complaints about isolated acts of false application practiced in Brazilian territory.

A highlight obtained in the satellite unit refers to the percentage of “lost” doses, similar to that found in Belgium^(5)^. Moreover, reports on wasted doses due to the “dead space” of syringes, mentioned in this study, were also found in experiences from European Union countries^(5)^, indicating the need for planning (including the purchase of inputs) to be carried out in an intersectoral manner.

It is noteworthy that the scarcity of different components in the supply chain and its impact on vaccine supply, at a global level, has culminated in strict control at different levels, in addition to the inclusion of numerous efforts and strategies in services, aiming to mitigate the loss of doses of vaccine. Among the best practices used to reduce wasted doses is the creation of waiting lists, a trend among countries, according to the latest report published by the European Center for Disease Prevention and Control^(18)^.

The vaccination campaign against COVID-19 has taken place in a national political scenario permeated by great obstacles and uncertainties. Although the WHO^(11)^ considers mass vaccination strategies as an adequate response to the pandemic context, its recognition as a key element for controlling the pandemic in Brazil, as well as actions aimed at immunizing the population, were slowly employed in the country^(19,20)^.

As a result, the national picture was one of shortages of inputs, problems of supply and production of vaccines as well as fragile operational strategies for implementing the campaign against COVID-19^(19,20)^. Thus, considering the description of this experience, it is expected to strengthen the debate, still limited, regarding the strategies for implementing the vaccination campaign against COVID-19 in emerging countries in scientific literature.

## CONCLUSION

The implementation of a drive-through vaccination satellite unit, during the campaign against COVID-19, required rigorous planning in addition to structural conditions, development of processes/flows and intense coordination between the team. This organization made it possible to serve a large audience, contributing to the vaccination coverage rate expansion in the city of São Paulo.

It should be noted that the planning of structure and processes stage, based on user and professional safety – a necessary characteristic in the face of the pandemic scenario –, was essential to achieve the results and ensure the vaccine use. In this sense, the fundamental and strategic role of professional nurses is highlighted in different points of action, from management to care.

Considering the lessons learned and reflections arising from this experience, some recommendations were outlined in order to help future projects. Regarding the structural aspects, a point of attention refers to the need to organize the cold chain close to the application sector as well as ensuring that the support unit (which stores and supplies vaccines) is easily and quickly accessible.

Another aspect to be considered concerns the introduction of technologies in the vaccination process, such as the use of tablets to record user information and the internet network for computerization and data sharing in real time. As for team establishment and organization, it is understood that ensuring a fixed team to carry out the activities is a factor to be considered for future experiences. It is also suggested to maintain leadership roles in the different sectors.

Furthermore, considering that the target audience and the actions were aimed at people with automated vehicles (cars and motorcycles), access to the unit by pedestrians was restricted. Thus, it is understood that offering options for vaccinating pedestrians in locations close to the drive-through can enhance and expand the window of opportunity.

Finally, in terms of scientific production, it is suggested the development of studies that explore parameters related to the time spent in the procedure of vaccine administration in large vaccination campaigns developed in scenarios similar to the drive-through model, to support future experiences with immunization team sizing.

## ASSOCIATE EDITOR

Cristina Lavareda Baixinho

## SUPPLEMENTARY MATERIAL

The following online material is available for this article:

### Chart 1.

Distribution of materials and supplies according to sector. São Paulo, SP, Brazil, 2021.

### Chart 2.

Description of activities performed by professionals according to role and work sector. São Paulo, SP, Brazil, 2021.
