# Supplementary material for: Implementation of the drive-through strategy for COVID-19 vaccination: an experience report
Source: Rev Esc Enferm USP. 2022 May 16;56:e20210397. doi: 10.1590/1980-220X-REEUSP-2021-0397en (PMC10353730; doi:10.1590/1980-220X-REEUSP-2021-0397en)
Supplement: Distribution of materials and supplies according to sector. São Paulo, SP, Brazil, 2021. [file 1980-220X-reeusp-56-e20210397-s1.pdf]

Rev Esc Enferm USP

https://doi.org/10.1590/1980-220X-REEUSP-2021-0397en

Supplementary Material to "Implementation of the drive-through strategy for COVID-19 vaccination: an experience report"

Chart 1 - Distribution of materials and supplies according to sector. São Paulo, SP, Brazil, 2021.

Application station			
Item	Qnt*	Item	Qnt*
Needle 25x7 or 30x7mm	30 un†	Disposable cups	2 un†
Alcohol gel	1 bottle	Sheet for bottle control	1 un†
Liquid alcohol	1 bottle	Preview map sheet	1 un†
Cotton	1 pack ‡	Table	1 un†
Aluminum tray	1 un	Disposable syringe 3 ml	1 un†
Blood stop	1 bx§	Swab	100 un†
Recyclable ice coils	3 to 4 un †	Maximum/minimum thermometer	1 un†
Chair	2 un†	Table: temperature control	1 un†
Polystyrene box	1 un†	Office supplies (pen, artboard, pencil, eraser)	1 un † (each)
Sharp cutting material collector	1 un†		

Emergency	
Item	Item
ASA 100 mg	Sterile gauze
Adenosine 3 mg	Glucose 50%
Needle 30x7mm	Glucose meter
Needle 40x12mm	Caps
Amiodarone 50 mg/ml (amp3 ml)	Hydrocortisone 100 mg
Bandage 10	Hydrocortisone 500 mg
Bandage 15	Jelco no. 18
Atropine 0.25mg/ml	Jelco no. 22
Guedel cannula no. 1	Jelco no. 24
Guedel cannula no. 3	Jelco no. 20
Intubation cannulas no. 4.0 (no cuff)	Curved blade no. 02
Intubation cannulas no. 6.0 (with cuff)	Curved blade no. 03
Intubation cannulas no. 6.5 (with cuff)	Curved blade no. 04
Intubation cannulas no. 7.0 (with cuff)	Curved blade no. 05
Intubation cannulas no. 7.5 (with cuff)	Straight blade no. 01
Intubation cannulas no. 8.0 (with cuff)	Straight blade no. 02
Intubation cannulas no. 8.5 (with cuff)	Straight blade no. 03
Clopidogrel 75mg	Straight blade no. 04
Sodium chloride 0.9% 10 ml	Laryngoscopy
Automated external defibrillator	Gloves 7.5
Diazepam 5 mg/ml	Gloves 8.0
Dipyrone 500 mg	N95 mask
Dipyrone drops	Midazolam 5 mg/ml
Epinephrine 1mg/ml	Safety goggles
Phenytoin	Paracetamol drops
HePA/HMEF filter	Laryngeal battery (in casing)
Adult guide wire	Plate
Child guide wire	Salbutamol 100 mcg
Adhesive tape	Syringes 20 ml
Endotracheal tube fixator	Saline 0.9%
Tourniquet	Glycosate serum 5%
Gauze	Xylocaine spray

*Qnt= quantity; †Un = unit; ‡Pack= package; §Bx= box
