# Supplementary material for: Implementation of the drive-through strategy for COVID-19 vaccination: an experience report
Source: Rev Esc Enferm USP. 2022 May 16;56:e20210397. doi: 10.1590/1980-220X-REEUSP-2021-0397en (PMC10353730; doi:10.1590/1980-220X-REEUSP-2021-0397en)
Supplement: Description of activities performed by professionals according to role and work sector. São Paulo, SP, Brazil, 2021. [file 1980-220X-reeusp-56-e20210397-s2.pdf]

https://doi.org/10.1590/1980-220X-REEUSP-2021-0397en

Supplementary Material to "Implementation of the drive-through strategy for COVID-19 vaccination: an experience report"

Chart 2 - Description of activities performed by professionals according to role and work sector. São Paulo, SP, Brazil, 2021

Sector	Role	Professional	Description of activities
SCREENING	Screening professional	Community health worker	Guide users on the eligibility criteria of priority groups and contraindication (according to the Municipal Health Department instructions)
SCREENING	Screening and registration leader	Oral health assistant, dental surgeon or multidisciplinary team	Update screening team as the latest instructional criteria
SCREENING	Screening and registration leader	Oral health assistant, dental surgeon or multidisciplinary team	Clarify questions from the screening team
SCREENING	Screening and registration leader	Oral health assistant, dental surgeon or multidisciplinary team	Share team questions with the coordinator
SCREENING	Screening and registration leader	Oral health assistant, dental surgeon or multidisciplinary team	Clarify users' doubts about inclusion criteria
SCREENING	Screening and registration leader	Oral health assistant, dental surgeon or multidisciplinary team	Assist in monitoring and organizing the breakfast and lunch schedule
FLOW ORGANIZATION	Flow organizer	Environmental promotion worker or community health worker	Organize cart and queue flows
FLOW ORGANIZATION	Flow organizer	Environmental promotion worker or community health worker	Direct carts to available app units
FLOW ORGANIZATION	Flow organizer	Environmental promotion worker or community health worker	Avoid on-site accidents
USER REGISTRATION	Screening and registration leader	Dental surgeon or Oral health assistant or multidisciplinary team	Responsible for the preview of tablets
USER REGISTRATION	Screening and registration leader	Dental surgeon or Oral health assistant or multidisciplinary team	Lead contingency plan in case of systemfall (manual record sheet for later posting)
USER REGISTRATION	Screening and registration leader	Dental surgeon or Oral health assistant or multidisciplinary team	Contact IT staff when needed
USER REGISTRATION	Screening and registration leader	Dental surgeon or Oral health assistant or multidisciplinary team	Request more resources, according to demand
USER REGISTRATION	Screening and registration leader	Dental surgeon or Oral health assistant or multidisciplinary team	Control the charge level of tablets' batteries
USER REGISTRATION	Screening and registration leader	Dental surgeon or Oral health assistant or multidisciplinary team	Check the batch of vaccine used on the day with the registration in the pre-filled vaccination vouchers
USER REGISTRATION	Screening and registration leader	Dental surgeon or Oral health assistant or multidisciplinary team	Clarify registrants' doubts
USER REGISTRATION	Screening and registration leader	Dental surgeon or Oral health assistant or multidisciplinary team	Withdraw the vaccination vouchers
USER REGISTRATION	Screening and registration leader	Dental surgeon or Oral health assistant or multidisciplinary team	Train the team for registration in the information system
USER REGISTRATION	Registration organizer	Oral health team or multidisciplinary team	Register in the information system
USER REGISTRATION	Registration organizer	Oral health team or multidisciplinary team	Carry out contingency plan when the system falls
USER REGISTRATION	Registration organizer	Oral health team or multidisciplinary team	Check if the user is from the eligible group
USER REGISTRATION	Registration organizer	Oral health team or multidisciplinary team	Deliver pre-filled vaccine voucher
COLD CHAIN AND APPLICATION	Applicator	Nursing assistant	Guidance
COLD CHAIN AND APPLICATION	Applicator	Nursing assistant	Control the thermal boxes' temperature
COLD CHAIN AND APPLICATION	Applicator	Nursing assistant	Request replacement of table material and supplies
COLD CHAIN AND APPLICATION	Applicator	Nursing assistant	Aspirate and apply the immunobiological agent according to the appropriate technique
COLD CHAIN AND APPLICATION	Applicator	Nursing assistant	Report adverse events or vaccination failures, immediately after vaccination
COLD CHAIN AND APPLICATION	Applicator	Nursing assistant	Record information on the user's card and control sheet
COLD CHAIN AND APPLICATION	Support professional	Nursing assistant or multidisciplinary team or oral health team	Offer support for performing the procedure (tray support)

Sector	Role	Professional	Description of activities
			Record information on the map, vaccination card
	Application supervisor	Nurse	Ensure: delivery and use of personal protective equipment, cold chain of stations, proper technique of the procedure
			Lead: organization, replacement of vaccine, materials and station supplies, cold chain (temperature control, stock of vaccine and supplies), supply of stations and pre-filling of vaccination certificates
			Inform, after closing, the total number of doses left to the basic support unit, to start the call of the waiting list while they are transported
	Cold chain and application leader	Senior nurse	Lead the team in table assembly
			Lead the team in thermal box assembly
			Technical responsible for the nursing team
			Conduct the training of nurses
			Clarify questions about application and users' questions
			Responsible for previews
			Perform the lunch and breakfast schedule of the nursing team
			Receive vaccines
			Arrange the nursing team at the tables
			Ensure the closure of the sharps and infectious agents shelter gate after dismantling the vaccine application area
			Perform safety huddle safety at the beginning of duty
	Emergency leader	Physician	Make notification of post-vaccination events
			Attend to emergencies
			Clarify users' doubts about vaccine contraindication
			Check the emergency cart
			Request replacement of emergency cart material
			Emergency key storage and control
			Decision making whether the immunobiological agent would be applied on site or if the user would be referred to the Basic Health Unit
	Cold chain assistant	Nursing assistant	Responsible for checking, with the stations' assistants, the temperature of the boxes every hour
			Control exchanges of bottles filled with voids
			Check the need for refueling of vaccine at stations
			Ensure vaccine safety by avoiding temperature change, loss and waste
			Monitor the transport of vaccines between the satellite unit and the Basic Health Unit (at the beginning and end of the day)
			Ensure the correct storage of sharps and infectious agents, in the shelter intended for this purpose
ADMINISTRATION	Administrative support	Technical or administrative support	Lead material inventory count
			Request replacement
			Lead meal receipt and control
			Ensure water replacement
			Ensure that there is no food left in the snack and lunch cooler, distributing them all on the same day they were received
			Control and storage of locker keys, material storage and dining area
			Tablet control
			Presence control
	Coordinator	Nurse coordinator/institutional technical supporter	Perform the work schedules of the different sectors, requesting the presence of professionals from different services
			Follow the stadium opening and closing routine due to games to align the schedule
			Monitor the update of vaccination instructions, disseminating information and training leaders and teams on the team change day

Sector	Role	Professional	Description of activities
			Report and participate in the solution of infrastructure, personnel, materials, logistics, cold chain, immunobiological agents, etc.
			Monitor the work routine of all drive-through sectors, mediating conflicts and observing the work, seeking excellence in processes
			Perform dialogue between the different instances, referring to the activities developed
			Ensure the closure of the sharps and infectious agents shelter gate after dismantling the vaccine application area
	Manager	Nurse	Conduct support, dialogue and support for media and press
			Food
			Transportation
			Acquisition or loan of goods
			Conflict mediation
			Search for excellence in processes
